# Therapeutic Benefits and Dietary Restrictions of Fiber Intake: A State of the Art Review

**DOI:** 10.3390/nu14132641

**Published:** 2022-06-26

**Authors:** Corina-Bianca Ioniță-Mîndrican, Khaled Ziani, Magdalena Mititelu, Eliza Oprea, Sorinel Marius Neacșu, Elena Moroșan, Denisa-Elena Dumitrescu, Adrian Cosmin Roșca, Doina Drăgănescu, Carolina Negrei

**Affiliations:** 1 Department of Toxicology, Faculty of Pharmacy, “Carol Davila” University of Medicine and Pharmacy, 3-6, Traian Vuia Street, Sector 2, 020945 Bucharest, Romania; corina-bianca.ionita-mindrican@drd.umfcd.ro (C.-B.I.-M.); carolina.negrei@umfcd.ro (C.N.); 2 Department of Clinical Laboratory and Food Safety, Faculty of Pharmacy, “Carol Davila” University of Medicine and Pharmacy, 3-6, Traian Vuia Street, Sector 2, 020956 Bucharest, Romania; elena.morosan@umfcd.ro; 3 Microbiology Department, Faculty of Biology, University of Bucharest, 1-3 Portocalilor Way, 060101 Bucharest, Romania; 4 Professional Farma Line, 116 Republicii Street, 105200 Baicoi, Romania; sorinel.neacsu@pfarma.ro; 5 Department of Organic Chemistry, Faculty of Pharmacy, “Ovidius” University of Constanta, 6, Căpitan Aviator Al Șerbănescu Street, 900470 Constanta, Romania; denisa.dumitrescu@univ-ovidius.ro; 6 Department of Drug Analysis, Biopharmacy and Biological Medicines, Faculty of Pharmacy, “Ovidius” University of Constanta, 6, Căpitan Aviator Al Șerbănescu Street, 900470 Constanta, Romania; cosmin.rosca@univ-ovidius.ro; 7 Department of Pharmaceutical Physics and Informatics, Faculty of Pharmacy, Carol Davila University of Medicine and Pharmacy, 6, Traian Vuia Street, 020956 Bucharest, Romania; doina.draganescu@umfcd.ro

**Keywords:** dietary fiber, metabolic disorders, health effects, dietary fiber characterisation, metabolic syndrome, type 2 diabetes, dyslipidaemia, short-chain fatty acids

## Abstract

Throughout history, malnutrition and deficiency diseases have been a problem for our planet’s population. A balanced diet significantly influences everyone’s health, and fiber intake appears to play a more important role than previously thought. The natural dietary fibers are a category of carbohydrates in the constitution of plants that are not completely digested in the human intestine. High-fiber foods, such as fruits, vegetables and whole grains, have consistently been highly beneficial to health and effectively reduced the risk of disease. Although the mode of action of dietary fiber in the consumer body is not fully understood, nutritionists and health professionals unanimously recognize the therapeutic benefits. This paper presents the fiber consumption in different countries, the metabolism of fiber and the range of health benefits associated with fiber intake. In addition, the influence of fiber intake on the intestinal microbiome, metabolic diseases (obesity and diabetes), neurological aspects, cardiovascular diseases, autoimmune diseases and cancer prevention are discussed. Finally, dietary restrictions and excess fiber are addressed, which can cause episodes of diarrhea and dehydration and increase the likelihood of bloating and flatulence or even bowel obstruction. However, extensive studies are needed regarding the composition and required amount of fiber in relation to the metabolism of saprotrophic microorganisms from the enteral level and the benefits of the various pathologies with which they can be correlated.

## 1. Introduction

From a quantitative point of view, to ensure a daily energy intake of 2000 kcalorie, a typical food macronutrient distribution should be provided by 250–275 g carbohydrates (from which 30–40 g of fiber), 100–125 g protein and under 66.66 g of primarily unsaturated fats [1]. An optimal amount of fiber can be obtained by the daily consumption of vegetables, legumes, whole grains and oilseeds in the diet. These foods are rich in essential nutrients (vitamins, minerals and antioxidants) and in fiber, thereby, providing all the benefits of a healthy and balanced diet. Nutritionists recommend that, for excellent digestion, a normal body weight and a low risk of developing cardiovascular diseases, at least 50% of all cereals consumed should be unprocessed. Most nutritionists recommend an intake of 18–38 g of fiber/day for adults, which is around 8–20 g per 1000 kcal [2,3,4]. The WHO/FAO and EFSA recommend an average daily intake of 25 g of fiber per adult [5,6].

A higher natural dietary fiber (NDF) intake is associated with fewer metabolic diseases (obesity, diabetes and cardiovascular disease) and plays an essential role in intestinal health. Increased NDF intake causes various physiological effects, both locally in the gut and systemically. For example, NDF can significantly alter the intestinal environment, affecting the intestinal microbiome and influencing the intestinal barrier, gastrointestinal immune and endocrine responses, the nitrogen cycle and microbial metabolism. These changes associated with the gut can then alter the physiology and biochemistry of the other major organs in the management of nutrients and detoxification of the body (liver and kidneys) [7].

Fiber intake is associated with other lifestyle factors, such as the increased consumption of fruits and vegetables and exercise. In addition, high fiber diets are usually lower in fat and energy density and help to maintain a healthy body weight [8]. E. H. Hipsley first used the name dietary fiber in 1953 for non-digestible constituents of plant origin [9]. Dietary fibers are found naturally in food and can be added to food for nutritional and therapeutic roles simultaneously, such as functional or processed fiber. Thus, functional fibers are extracted from natural sources and added to processed foods [10].

NDF refer to edible plant parts or carbohydrate equivalents in the composition of edible plants that are resistant to digestion and absorption in the small intestine but can be fermented partially or entirely in the colon. Polysaccharides, oligosaccharides, lignin and various chemicals linked with these categories in plants fall under this group (waxes, phytates, saponins, tannins, etc.). The laxative action, cholesterol reduction and serum glucose reduction are vital physiological effects of these nutrients [11,12,13]. A classification of the dietary fibers obtained from food is presented below (Table 1).

There are two forms of dietary fiber based on their capacity to dissolve in water: soluble fibers (those that, in the presence of water, form colloidal solutions in the intestine, slow down digestion and absorption of nutrients, which provides a prolonged feeling of satiety and a decrease in appetite, such as and a reduction in food glycemic index) and insoluble fibers (those that pass primarily intact through the digestive system, accelerating intestinal transit and playing an important role in the body’s detoxification process). Soluble fibers includes gums, pectins, beta-glucans and oligosaccharides [22]. The richest sources of soluble dietary fibers are apples, pears, citrus fruits, carrots, broccoli, peas, cucumbers, celery and oat bran [23]. 

Insoluble dietary fibers include lignin, cellulose, hemicellulose, chitin, resistant starch and resistant dextrin [24,25], which have a laxative effect, are recommended by specialists to people suffering from constipation. Nuts, beans, whole wheat, barley and roots are the best sources of insoluble dietary fiber [26]. Soluble and insoluble dietary fibers are found in varying amounts in plant-based foods. The foods richest in fiber are cereal bran (wheat and oats), whole grains, legumes (lentils, beans) and dried fruits (plums, apricots). 

Due to the high water concentration, fresh fruits and vegetables provide relatively little fiber when this amount is related to fresh weight. On the other hand, these foods are relatively high in fiber when the amount of fiber per 100 kcal is reported [27]. Thus, cereals provide on average 36% to 65% of the daily fiber intake in industrialized countries, fruits 6% to 24%, leguminous 22% to 47% and green vegetables 2% to 8% [28,29].

This review is structured into nine paragraphs and describes the main influences of fiber from food on the human body, including its impact on metabolic diseases, digestive system, neurologic, cardiovascular, autoimmune diseases, cancer prevention and dietary fiber restrictions. The cited literature was selected using MEDLINE, Embase, Web of Science Core Collection and Google Scholar databases. The search terms included obesity and high fiber consumption, dietary fiber and metabolic diseases, fiber metabolism and health benefits, autoimmune disease and dietary fiber intake, evaluation of high-fiber diet and modern dietary practices, daily fiber intake and chronic disease.

## 2. Metabolism of Natural Dietary Fibers (NDF)

**Figure 1:**
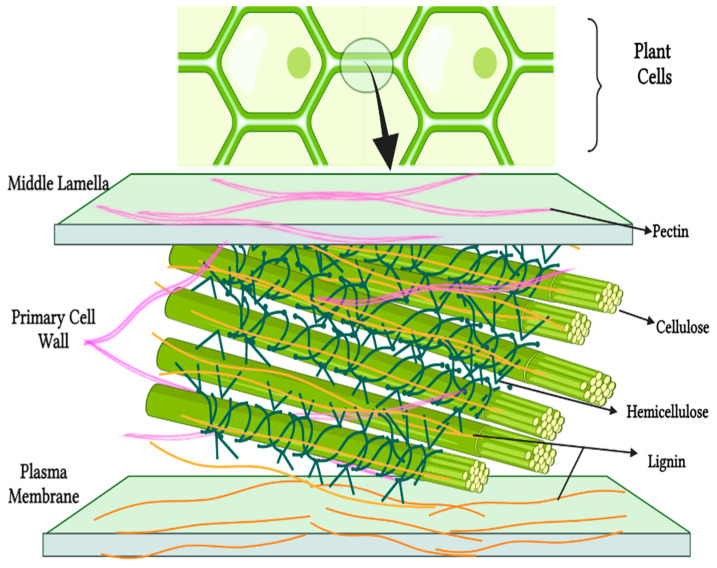
Fiber localization in plants. Created with BioRender.com.

NDF are represented by carbohydrates that are mainly part of the cell walls of plants (Figure 1), natural compounds that are not completely digested by the human intestine, in the category of gums, pectins, mucilages, cellulose, hemicellulose, lignin, etc.

NDF are the fraction of carbohydrates that remains undigested in the upper digestive tract. The bacteria metabolized the soluble, fermentable dietary fiber in the ileum and the ascending colon and the insoluble and high viscosity fiber are partially fermented in the distal colon, where the density of the microbiome is higher and the motility is lower. NDF degradation involves many microorganisms organized in the food chain. At the top of this food chain are fibrolytic bacteria. They degrade complex polysaccharides into oligosides and then into monosaccharides. 

At a lower level of this chain are the glycolytic bacteria that ferment the available ozone. Intermediate products (lactate, formate, succinate, etc.) and/or final fermentation products are generated, short-chain fatty acids or SCFAs (acetate, propionate and butyrate), as well as gases (hydrogen and carbon dioxide). The acetate formed is absorbed and metabolized in peripheral tissues, where it is used as a precursor in cholesterol and fatty acid production. Propionate is a precursor to gluconeogenesis and is absorbed and processed in the liver. As a result, liponeogenesis and cholesterol production is also inhibited [30].

**Figure 2:**
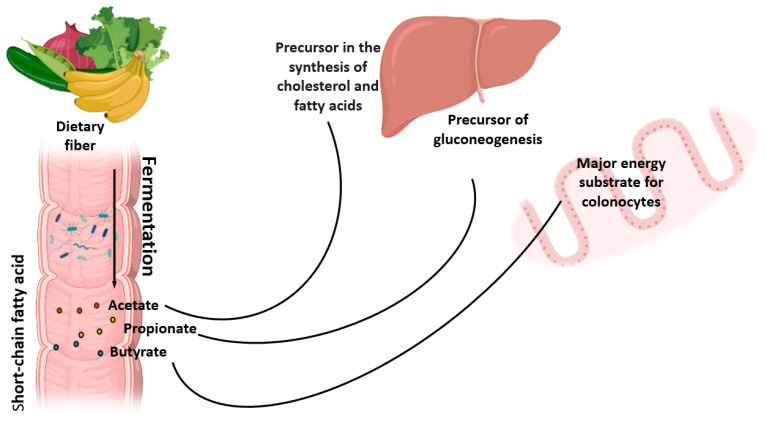
Fiber fermentation and its utilization pathways. Created with BioRender.com.

Therefore, propionate is proposed as a potential metabolite for preventing obesity and diabetes. Butyrate is metabolized by cells in the colon, and thus butyrate is a primary energy substrate for colonocytes and enterocytes, promoting the development of strains of *Bifidobacterium* sp., important probiotics for homeostasis of the human body (Figure 2). SCFAs also stimulates intestinal motility, transit and activates the production of satiety hormones [31,32,33].

The interaction of NDF with the intestinal microbiome depends on their physicochemical properties. Thus, the insoluble dietary fibers are very slightly fermented but stimulate the intestinal transit and reduce the fermentation time of the intestinal contents in the colon. Furthermore, the mechanism of action of insoluble dietary fiber is physical because it increases the stool bowl by increasing the degree of hydration and its volume (since insoluble dietary fibers are organized in the form of a matrix in which water accumulates) and intestinal emptying time decreases [34,35].

Psyllium seeds contain soluble fiber but with high viscosity, which ferments little. They have a high capacity for water absorption and gelling, forming a viscous gel in the intestinal lumen, thus, preventing the absorption of cholesterol, glucose and the reabsorption of bile salts. They have the effect of stabilizing serum cholesterol level by inhibiting its intestinal absorption and by stabilizing the secretion of insulin, which promotes hepatic cholesterol synthesis. Increased intestinal volume fosters a feeling of satiety and reduced appetite [36].

Through intestinal fermentation, soluble dietary fibers with a reduced degree of viscosity (pectins and fructo-oligosaccharides) promote the production of SCFAs, including acetic, propionic and butyric, as well as carboxylic acids, such as lactic acid [37,38]. SCFAs have been found to operate as signaling molecules by binding to the FFA2, FFA3, GPR109a and Olfr78 membrane receptors in the intestine [39,40].

FFAR2 regulates the energy homeostasis of the whole body by modulating lipid differentiation and lipid storage in adipocytes and by regulating the production of leptin. This intestinal hormone inhibits the sensation of hunger in the CNS. SCFA-activated FFAR2 mediates the activation of the intestinal immune and anti-inflammatory response by producing cytokines and chemokines [41,42].

There are clinical studies in laboratory rats that showed that animals fed a high-fiber diet had a lower increase in body mass compared to those fed a standard diet high in carbohydrates (starch) and that animals with a supplemented diet with propionate and butyrate showed a better glucose tolerance compared to those fed a standard diet. Similarly, insulin tolerance was significantly improved in animals on a fiber-supplemented diet or in butyrate and propionate [43,44].

## 3. Natural Dietary Fibers and the Intestinal Microbiome

The gastrointestinal tract microbiota in humans is composed of bacteria, fungi, archaea, protozoans and viruses. More than 90% of the 12 different phyla are *Proteobacteria*, *Firmicutes*, *Actinobacteria* and *Bacteroidetes*, while the main “enterotypes” are from genus *Bacteroides*, *Ruminococcus* and *Prevotella* [45]. Dysbiosis, or imbalances in the microbiota’s composition, hae been linked to digestive (Crohn’s disease, irritable bowel syndrome, etc.), metabolic (obesity, diabetes, etc.) and other disorders (allergies, autism, etc.) [46,47].

Although these disorders are likely complex, the current research increasingly views the microbiome as a component that should not be overlooked when preventing or curing some of these diseases. Current research strategies focus on, among other things, the use of probiotics and/or prebiotics. The microbiota degrades the dietary fiber, releasing a sequence of antioxidant and/or anti-inflammatory chemicals bioavailable to the host. Thus, the colonic microbiota’s breakdown of NDF has a role in avoiding a variety of illnesses, including digestive (colorectal cancer, colitis and infections) and metabolic disorders (diabetes, cardiovascular diseases and obesity) [48], whether it is the strengthening of the immune system affected by various factors, including drugs [49,50].

Dietary fiber consumption has decreased, while sugar and animal protein consumption has increased, and microbial diversity in the human gut microbiome has decreased. These events can affect the microbiome’s function and the creation of SCFAs, leading to the development of chronic inflammatory illnesses [51].

Prebiotics are dietary fibers that specifically encourage the development and/or activity of gut bacteria that may be linked to the health and well-being of the host. They reduce the prevalence and duration of infectious or antibiotic-associated diarrhea, reduce symptoms associated with inflammatory diseases of the digestive tract, exert a protective effect against colorectal cancer, reduce the risk of cardiovascular disease, increase satiety, weight loss and thus prevent obesity, promote mineral bioavailability (calcium, magnesium and iron) and reduce allergies [48].

Short-chain carbohydrates work as prebiotics, promoting the growth of beneficial bacteria in the intestine. For example, inulin is a water-soluble fructose polymer with a polymerization degree of 2 to 60. Inulin undergoes fermentative oxidation in the small intestine’s terminal segment and the colon, resulting in the creation of SCFAs. As a result, they stimulate the growth of *Bifidobacterium* strains, which are vital probiotics for maintaining human body homeostasis [52].

Propionic acid is absorbed into the portal vein and reaches the liver, where it is metabolized by gluconeogenesis. It also activates FFAR2 receptors, with a role in stimulating the immune system. In addition, some studies confirmed the inhibition of hepatic cholesterol synthesis in the presence of propionate [53].

Colonocytes use butyric acid as an energy substrate. Butyric acid is involved in modulating the growth of intestinal epithelial cells, with benefits in the prevention and treatment of colon cancers. The species of butyrogenic bacteria are *Faecalibacterium prausnitzii* and *Eubacterium rectale*. Butyric acid is an energy source for digestive tract cells. This was evidenced by the comparative measurement of ATP produced by simple colonocytes and colonocytes populated with bacteria specific to the gut microbiome. Unpopulated murine intestinal cells with bacteria had a NADH/NAD + ratio that was 16 times lower than homonymous cells populated with intestinal bacteria. The resulting ATP level was also 56% lower [54].

Butyrate promotes epithelial growth in the colon but, paradoxically, has an inhibitory effect on colorectal cancers. This phenomenon is explained by the characteristic Warburg effect of cancer cells. Non-cancerous colonocytes use oxygen to produce energy (aerobic glycolysis), while cancerous colonocytes produce energy by anaerobic glycolysis, even if oxygen is available. The use of pyruvate and butyrate as an energy substrate also decreases in the affected cells. Butyric acid accumulates in the cytoplasm and acts as an HDAC (histone deacetylases) inhibitor, which reduces the underlying mechanisms of cellular apoptosis [55].

Soluble dietary fibers play an important role in balancing the gut microbiome (through the proliferation of intestinal bacteria of the species belonging to the genera *Eubacterium*, *Bifidobacterium* and *Lactobacillus*), which is why it is recommended in both the treatment of constipation and the treatment of diarrhea. Studies on the intestinal microbiome are numerous, with the little data currently available. However, the role of the microorganisms in achieving optimal digestion in the synthesis of vitamin K and the absorption of certain minerals or even medicinal substances (digoxin) is known. In addition, some studies relate the gut microbiome to the immune system functions (FFAR2 activation of SCFAs) and to the central nervous system health (the relationship between intestinal microbiome and depression, as a result of serotonergic depletion, was described) [56].

According to clinical investigations in rats, intestinal colonization varies depending on the kind of fiber ingested. For example, laboratory mice fed on diets containing 5–10% cellulose showed a significantly different bacterial community than mice fed a diet with 10% fructo-oligosaccharide (FOS) or inulin [31,57]. Cross-feeding is a fascinating phenomenon that occurs in the gut in the dietary fiber presence: the breakdown of products from one bacterial species’ fermentation of a complex carbohydrate become a substrate for the activity of another bacterial species. *Bifidobacteria* and *Lactobacillus*, for example, utilize fructans as a source of energy. Lactic acid and propionic acid are produced during the fermentation of fructans. *Eubacterium*, *Roseburia* and *Faecalibacterium* use it as an energy source when converting lactate and propionate to butyric acid [31].

Soluble dietary fibers also have a physical mechanism of action with an important role in modulating the absorption of certain macro and micronutrients. For example, due to their high molecular weight and water solubility, they increase the viscosity of the intestinal contents, preventing the absorption of cholesterol and glucose and the reabsorption of bile salts. This stabilizes serum cholesterol levels, by inhibiting intestinal absorption and stabilizing insulin secretion, which promotes hepatic cholesterol synthesis. In addition, increasing the intestinal volume promotes the feeling of satiety and reduces appetite [30].

Whole grains, vegetables and fruits high in fiber are considered the richest foods in prebiotic components. In addition, prebiotics can stimulate increased gastrointestinal balance and the growth and metabolism of healthy bacteria in the intestinal tract. Probiotics and prebiotics play an important role in forming of the gut microbiome by affecting immune system development, decreasing inflammation and oxidative stress and creating a balanced immune response that protects against pathogen colonization. In addition, a well-balanced diet promotes the growth of a healthy gut microbiome capable of producing SCFAs, which act as signaling molecules that influence the immune system’s proper functioning and promote cell apoptosis by inhibiting cancer cell development and proliferation [58].

Honey is also a potential source of prebiotic components as well as other bee products (bee-pollen [59], bee-bread [60], propolis [61], etc.). Honey oligosaccharides have a potential prebiotic activity by stimulating the growth of beneficial microorganisms (*Lactobacillus* sp. and *Bifidobacterium* sp.): also, most of the antioxidant compounds in honey stimulate their growth. Due to its complex composition, honey may contain probiotic microorganisms and prebiotic components, and the osmotic constitution of honey may have a protective role for probiotic bacteria in the gastrointestinal tract. An 821-week study of malnourished children found that honey intake led to increased plasma levels of short-chain fats [62].

According to a 2019 study done in Brazil on several kinds of honey, the prebiotic impact enhances as the fructose level increases [63]. In a study comparing honey with prebiotics, such as inulin, galacto-oligosaccharide (GOS) and FOS, on *Bifidobacterium* cultures (*Bifidobacterium breve*, *Bifidobacterium adolescentis*, *Bifidobacterium longum* and *Bifidobacterium infantis*), it was discovered that lactic and acetic acids increased the production.

The prebiotic effect of honey is similar to that achieved by GOS, FOS and inulin by the proliferation of intestinal bacteria. Implicitly, the formation of acids, such as lactic and acetic, and the reduction of the intestinal pH can be explained by the significant concentration in honey (4–5%) of oligosaccharides [64]. In addition, reducing the intestinal pH inhibits the growth and development of Gram-negative pathogenic bacteria [58].

**Figure 3:**
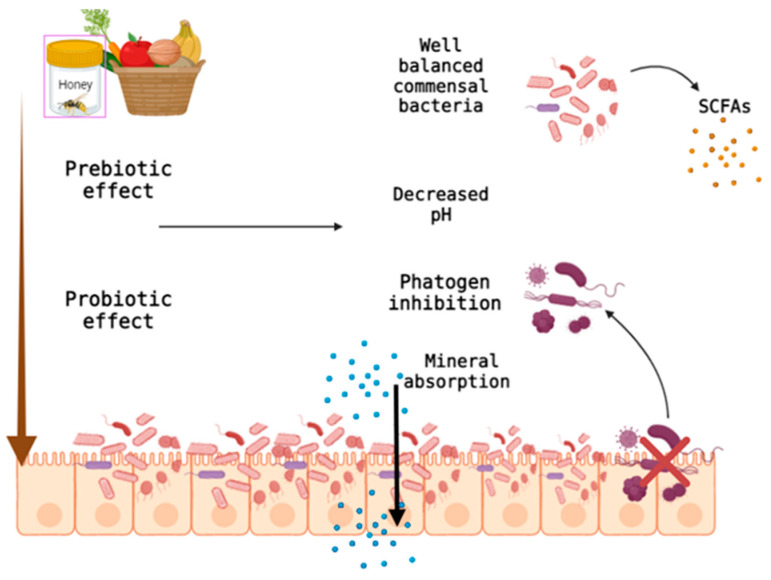
Prebiotic effect of honey and unprocessed fiber on the digestive system. Created with BioRender.com.

As with vegetable fiber, honey has a prebiotic effect ensuring good functioning of the digestive system (Figure 3). In addition to colonizing and growing beneficial microorganisms, honey can modulate oxidative stress, stimulating cell apoptosis and reducing cell proliferation [65]. These effects occur through various mechanisms (activation of the mitochondrial pathway, stopping the cell cycle and increasing the permeability of mitochondria) [66].

**Figure 4:**
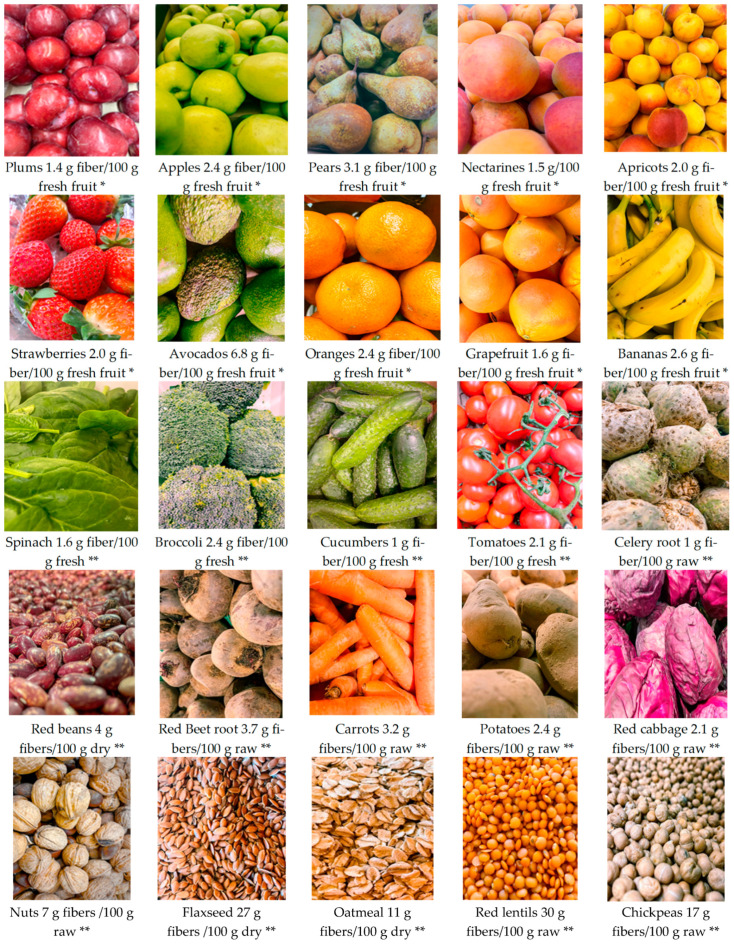
The estimated fiber content of certain vegetable products *—[67] and **—[68].

The intestinal tract health can be supported both by introducing fiber to the diet and by the moderate consumption of honey. Figure 4 shows different vegetables, fruits and seeds that are high in fiber.

Epidemiological studies suggest a two-way link between type 2 diabetes and depression. These diseases are induced by a high-fat and high-sugar diet (HFHS) in mice. Supplementation of the HFHS diet with FOS protects against the development of diabetes by inducing intestinal gluconeogenesis (NGI).

Similarly, through NGI, SCFAs generated by the gut microbiota from soluble dietary fiber decrease the risk of type 2 diabetes and obesity. In the case of SCFAs, for propionate, the mechanism involves the prior activation of the free fatty acid receptor in the portal nerves and a reflex arc that initiates NGI, which explains the metabolic benefits of soluble dietary fiber. However, the effects are significantly amplified in HFHS-fed mice, which dramatically resist the development of obesity and maintain a normal tolerance to glucose and insulin. In particular, the FOS diet increases the abundance of *Prevotella intestinalis* bacteria while reducing *Firmicutes* bacteria [69,70,71,72].

Some authors believe that the greater the diversity of fiber in diets, the more diverse and even healthier the microbiota that is expected [73]. However, patients with inflammatory bowel (and general microbial dysbiosis) were shown to have a comparable capacity for dietary fiber fermentation and SCFAs release as healthy controls. Thus, at least in the case of patients with inflammatory bowel, the reinstating of a healthy microbiome status based only on a high fiber diet alone appears to be not applicable [74].

## 4. Dietary Fiber Consumption in Different Countries

It is known that the Western countries’ industrialization has gradually changed the diet, and the fiber intake is marginal. Thus, the increase in daily caloric intake, the refining of flours and the reduction in bread consumption rich in fiber (for instance, with oat, granola, barley and wheat) are the main factors that led to the decline in fiber consumption. Each country has its dietary characteristics, some diets are considered healthy, such as the Japanese and Mediterranean diets, while others, such as Western diets, are high in saturated fats, and foods high in sugar and low in fiber are considered unhealthy.

According to a survey published in 2005–2006, Germany had the highest average fiber intake among western states, with women consuming 23 g per day and men consuming 25 g per day. Other nations follow behind, with average daily intakes of 15 g for women and 20 g for men [16]. The French had an unusually high-fiber diet only a century ago. However, the typical fiber consumption in France is presently between 15 and 22 g per day, which is below the ideal range suggested by nutritionists [28].

In Romania, the consumption of vegetables, fruits, fish and dairy products is far below the nutritional recommendations, which means that the Romanian diet has a lower intake of necessary nutrients, such as calcium, potassium, vitamin D, polyunsaturated fatty acids and fiber, thus, leading to reasons of concern for the long-term health of the population [75]. A study in 2017 found that, in the autumn season, the Romanian adults eat, on average, only 9–10 g of fiber per day [76]. During the COVID-19 pandemic, there has been a growing interest in purchasing vegetables and fruits; however, the adult population is still far from reaching the recommended intake of specialists. Observational studies conducted during the pandemic period found that most respondents are accustomed to consuming only one serving of vegetables and fruits per day [77,78].

Fibers promotion in the French diet is ensured by messages that recommend increasing the consumption of foods rich in complex carbohydrates, such as whole grains or even fruits and vegetables [79]. This strategy has also been adopted internationally by the WHO [80].

In the US, fiber consumption is much lower. Observational studies have shown that about 5% of Americans consume sufficient fiber [81,82] according to the recommendations of American nutritionists: 14 g of fiber per 1000 kcal or 25 g/day for women and 38 g/day for men [83]. Low fiber intake associated with an increased intake of saturated fat is considered as one of the leading causes of the increased incidence of cardiovascular disease in the US compared to other countries, such as Japan and Mediterranean countries [84].

The traditional Japanese diet is characterized by a high intake of rice, vegetables and fruits but also protein from fish and soy. A study that looked at the Chinese diet compared to the Mediterranean, Japanese and American diets showed that the Chinese diet had a lower daily intake of fiber, calcium, phosphorus and vitamins. In recent years, the development of the economy has led to the transformation of this traditional Chinese diet, which was rich in fiber, into an unbalanced diet characteristic of the modern lifestyle. 

Observational studies have shown that the traditional diet has changed, currently, the intake of fruits and vegetables is insufficient, the intake of cereals has decreased, implicitly the intake of dietary fiber, while the intake of fats has increased dramatically, especially saturated. This nutrient imbalance is associated with an increase in diseases, such as diabetes, dyslipidemia, cardiovascular diseases related to high blood pressure, and some cancers and nutrient intake in the Chinese has become worse than in Americans [85].

In general, Mediterranean countries, such as Greece, Spain and Italy, with a tradition of high consumption of fruits, vegetables, olive oil, fish and seafood, are noted for a reduction in the mortality rate due to cardiovascular diseases [86,87,88]. However, the traditional Mediterranean diet is progressively being replaced by refined, low-nutrient foods, which may explain some of the population’s documented metabolic and nutritional issues. High consumption of saturated fats and refined carbs, poor fiber intake and sedentary behavior are unhealthy eating patterns [89].

Indians are recognized for their significant consumption of high-fiber foods and their Mediterranean diet. The results of a clinical trial done on a group of 1000 patients indicate the benefits of the Indo-Mediterranean diet in improving the health of individuals with severe coronary heart disease. A diet rich in cereals, fruits, vegetables and nuts, according to the study, lowers the risk of mortality from a heart attack in people at high risk of coronary heart disease [90].

The Indian diet is rich in unrefined grains and vegetable products; thus, achieving the required daily fiber intake is easy to achieve. However, depending on the socio-economic groups, the fiber intake varies between 15 and 41 g fiber/day, in women the intake is lower than 15–30 g per day, and in the case of the tribal population, the amount of fiber consumed per day is much lower 15–19 g [91].

## 5. Dietary Fibers and Metabolic Diseases

Metabolic disorders (MD) are a collection of risk factors for cardiovascular disease, type 2 diabetes, especially cardiovascular and total mortality. The modern literature review shows cohort and randomized controlled trials, including generalized meta-analysis and systematic reviews, which indicate the relationship between MD and the impact of un-fermentable, gelling and high viscosity soluble dietary fiber (especially from a supplemented diet) on body weight and fat content, allowing metabolic control, including the correction of metabolic disorders related to carbohydrates, lipids and purines, as well as the regulation of blood pressure [92].

Some studies reported a health improvement after fiber administration in humans (Table 2).

### 5.1. Obesity

Obesity is on the rise and is one of the biggest health problems that is beginning to affect more people, including the young population. Obesity pathology involves both genetic factors and unhealthy nutritional habits. In obesity, there is an imbalance in the energy balance. The diet of obese patients is unbalanced with multiple eating disorders. Two-thirds of obese people have a body mass index ≥35 kg/m^2^. The average calorie intake among men and women exceeds 4000 kcal/day in most cases (92%). 

The average cholesterol intake is below 300 mg/day; however, about 29% of patients have a high cholesterol intake exceeding 300 mg/day. Only 16% of obese people have a normal intake of the average protein intake. For NDF, the average daily dose is around 28–29 g/day; however, almost half of those who suffer from obesity have an insufficient intake (less than 25 g/day) [99]. A recent study shows that dietary fiber supplementation may limit liver production and storage of fat and cholesterol in the event of overeating [100].

Various medical and observational research has indicated that a high-fiber diet with consumption of at least 14 g/1000 kcal per day, as suggested by dietitians, plays a significant role in maintaining a healthy body weight [27,101]. Unfortunately, especially in industrialized nations, industrial growth has resulted in a rise in refined foods and a significant decline in the frequent consumption of high-fiber meals, increasing the occurrence of metabolic illnesses, including obesity and even among teenagers. The increased intake of vegetables, fruits, legumes and meals high in soluble fiber have been shown in clinical research to reduce body weight and body fat percentage [102].

The effects on body weight are exerted through several mechanisms [103,104]:

- the formation of a mechanical barrier at the intestinal level by increasing the viscosity, which causes a decrease in intestinal transit, a reduction in the absorption of glucose and fatty acids and a decrease in the percentage of adipose tissue;
- lowering the rate of glucose absorption and reducing the glycemic index of food, reducing the feeling of hunger;
- soluble dietary fiber influences the gut microbiome by growing saprotrophic bacterial species, having a prebiotic effect; and
- by fermentative degradation in the colon of the dietary fiber, saturated fatty acids are generated with short chains that contribute to the reduction of body weight by delaying the evacuation of the stomach, followed by the increase of the feeling of satiety and the reduction of insulin sensitivity.

Obesity is becoming more frequent among reproductive-age women. Weight increases exacerbate the loss of natural fertility. Abdominal obesity may boost the tendency to ovulatory problems and to the emergence of insulin resistance. Diet has an impact on ovulation. Ovulatory problems are relieved by moderate weight loss. According to cohort studies, a balanced diet of nutrients, fiber, plant proteins, minerals, vitamins and higher adherence to the Mediterranean diet is linked to a decreased incidence of infertility owing to ovulation abnormalities [105,106,107]. Diet is also given special care throughout pregnancy. 

Clinical studies have indicated that consuming sufficient NDF during pregnancy can help both the mother and the fetus. NDF prevents constipation, a major problem pregnant women face and decreases the risk of glucose intolerance and pre-eclampsia [108].

Fiber consumption during pregnancy provides several advantages, including preventing constipation, maintaining microbial diversity in the colon and lowering the risk of gestational glucose intolerance [109]. Therefore, pregnant women are recommended to consume 28 g of NDF per day in Australia. Unfortunately, as compared to pregnant women in Norway (32.2 g/day) and Denmark (28 g/day), Australian women had a lower than recommended fiber intake (24.1 g/day) during pregnancy. Low fiber consumption was also seen during pregnancy in the UK (17.2 g/day) and the USA (19.8 g/day) [108].

Oligo-fructose is a kind of natural dietary fiber that may be found in a wide range of fruits and vegetables (bananas, wheat, onions and garlic). This isa prebiotic fiber that acts as a food source for bacteria in the microbiota of the intestine. These fibers aid in the digestion of food and eliminates unwanted germs. As a result, investigations reveal that the intestinal microbiota of rats treated with oligo-fructose is comparable to that of weak animals. In overweight and obese adults and obese rats, dietary oligo-fructose reduced ghrelin secretion and increased tyrosine (PYY) peptide secretion. Ghrelin is a hormone produced and released mainly in the stomach, and small amounts can be secreted by the small intestine, pancreas and brain. It is also called the “hunger hormone” because it plays a role in stimulating appetite and helps maintain fat deposits, thus, explaining the beneficial role that oligo-fructose has in obesity [110].

Bananas, cinnamon, onions, garlic, leeks, asparagus and parsley contain inulin, a soluble fiber. It is not digested in the stomach; instead, bacteria in the colon ferment it to SCFAs (propionate, acetate and butyrate), which triggers the production of appetite-suppressing hormones, such as glucagon-like peptide 1 (GLP-1). Supplementing with inulin-type fructans (ITF) helps decrease harmful cholesterol (LDL). Supplementing with ITF has been shown to assist persons with type 2 diabetes to decrease their fasting blood glucose and increasing their good cholesterol (HDL). In addition, supplementing with inulin can help with cholesterol and glucose metabolism [111].

Clinical trials in laboratory mice have shown increased plasma GLP-1 and inhibition of ghrelin following a diet supplemented with FOS, inulin or pectin [112,113]. Insoluble fermentable dietary fiber, such as resistant starch, allow the production of GLP-1 and PYY over time, regulating long-term satiety in healthy and diabetic volunteers [114]. Supplementing the diet with soluble fiber over 10 g/day, such as FOS, inulin, psyllium and pectin, as well as Guar gum (2.5 g/day) and β-glucan (3 g/day), increases postprandial concentrations of GLP-1, PYY and on the contrary, decreases those of ghrelin [115].

In recent clinical investigations, a diet high in pure soluble fiber has also been related to development of hepatocellular carcinomas. However, due to the creation of a substantial quantity of butyrate following the fermentation of inulin, these liver tumors only developed in laboratory mice with intestinal dysbiosis. These findings suggest that consuming more soluble fiber in supplements has negative impacts [116].

### 5.2. Diabetes

Clinical studies have shown a significant reduction in insulin resistance in patients whose diet has been supplemented with fiber and foods high in fiber are recommended for diabetes, as fiber slows down the absorption of carbohydrates, preventing increased blood sugar critical threshold [117]. NDF reduces the glycemic index of foods, especially soluble ones, by forming a gelatinous mass that slows down food digestion, thus, causing a feeling of satiety and attenuation of postprandial hyperglycemia.

The mechanisms by which fiber influences satiety are multiple. For example, increased chewing time promotes the production of saliva and gastric acid, which can increase gastric distension. Some dietary fibers bind water, which can also increase distension. Finally, stomach distension is thought to trigger vagal signals related to satiety, which probably contribute to increased satiety during and after meals. 

In addition, certain dietary fibers can slow down gastric emptying and lower the rate of glucose absorption in the small intestine. When glucose is released slowly, the insulin response may also be slowed down. Slow and steady responses to postprandial glucose and insulin are sometimes associated with increased satiety [118,119]. NDF is suggested to help manage and prevent type 2 diabetes. According to scientific data from clinical and experimental research, increased intakes of high-fiber foods result in a considerable drop in blood glucose in people of diabetes [120,121].

However, there is debate about the role of a high-fiber diet in type 2 diabetes management due to a lack of knowledge about the exact amount and source of fiber required to enhance their glycemic index. Some studies suggest that not increased fiber intake might promote a drop in glycemic levels in overweight individuals with type 2 diabetes, rather caloric limitation followed by weight loss is recommended [122,123].

NGI, which produces a central nerve signal that effectively modulates brain-controlled energy balance measures, including appetite and fullness, has been the focus of clinical research on the function of NDF consumption in carbohydrate metabolism, fat and body weight, hepatic glucose production, insulin secretion and insulin sensitivity. NGI is a newly identified function that influences energy homeostasis. Interestingly, NGI can be induced by specific macronutrients, such as proteins or fermentable dietary fiber. Soluble dietary fiber are not digested by mammalian intestinal enzymes and takes time to pass from the small intestine to the distal intestine, where it stimulates NGI through the complementary effects of several metabolic products: SCFAs, such as butyrate, propionate and succinate. 

Butyrate activates the expression of NGI genes in enterocytes by increasing ATP, which activates adenylate cyclase through a substrate effect [124,125]. This promotes an increase in cyclic adenosine monophosphate (cAMP), the intracellular messenger that activates gene expression for gluconeogenesis. Finally, propionate and succinate serve as glucose precursors for NGI [126,127]. Therefore, food fermentable fiber acts as a reservoir of substrates for gluconeogenesis, depending on their fermentation by the intestinal microbiota. Interestingly, this tank may persist, as the so-called insoluble dietary fiber, with a high molecular weight, can also be partially fermented, although less quickly and at a more distal point than the fermentable dietary fiber.

In this situation of portal glucose release, there is a substantial improvement in the key functions of homeostasis of carbohydrates and energy. These improvements include decreased dietary intake, body weight, fat storage, decreased hepatic glucose production, increased insulin sensitivity and improved glucose metabolism [128].

Detection of glucose produced by NGI is necessary for these metabolic benefits, as no beneficial effects are observed in mice with genetically inactivated intestinal gluconeogenic function (by targeted elimination of intestinal G6Pase) or in the rat portal vein [128,129]. Thus, even when fed a standard diet, NGI-deficient animals have uncontrolled glycemic, including moderate fasting hyperglycemia and hyperinsulinemia, glucose intolerance and insulin resistance and insulin secretion and inability to respond to glucose. The SCFAs have been blamed for the positive benefits of soluble dietary fiber [40].

Type 2 diabetes leads to a decrease in the postprandial response to glucose and an increase in glycated hemoglobin (HbA1c). Several reviews and meta-analysis articles studied the effect of NDF (type and quantity) on glycemic control in obese, overweight and type 2 diabetics. In a longitudinal analysis of 17 prospective cohorts, an inverse relationship was found between dietary fiber (total dietary fiber, cereal fiber, fruit fiber or insoluble fiber over 25 g/day) and the risk of diabetes [130]. 

Numerous studies indicate an improvement in glycemic and HbA1c control in people with type 1 or type 2 diabetes who have consumed more dietary fiber. A meta-analysis over 8–16 weeks showed that eating high-fiber foods or supplementing with soluble fiber can reduce HbA1c and fasting plasma glucose levels in people with diabetes [131]. If we stratify dietary fiber, we realize that, regardless of the type of fiber, soluble (psyllium, β-glucan, inulin, FOS) [132,133,134] or insoluble (cellulose, resistant starch), they allow a decrease in blood sugar and HbA1c [134,135]. Epidemiological studies demonstrate a negative correlation between whole-grain fiber intake and the risk of type 2 diabetes, especially for the Mediterranean diet high in whole grains, fruits and vegetables [120,132,136].

## 6. Dietary Fibers and Neurological Impact

The link between the gut microbiota and the neurological system is a crucial component of the intestine-cerebellum axis, which has recently gotten much attention. Butyric acid, produced when NDF are fermented, serves important functions in metabolism and mitochondria. It might theoretically alter gene transcription by increasing acetylation. There have been no investigations on the metabolic effect of butyric acid in the intestines on the brain yet. However, the literature suggests that if sufficient butyrate levels could be obtained in the brain, it possibly serves as an energy substrate, similar to how it does in the colon and restore energy homeostasis; however, their exact concentration that affects the physiology of the brain through dietary changes, intestinal microbiome changes or pharmacological supplementation remains to be determined [137,138].

Butyrate has three main features in this context: (1) it directly affects energy metabolism by acting as a beta-oxidation substrate, (2) it activates genes associated with mitochondrial biogenesis (e.g., PGC1) by acting as a selective inhibitor of HDAC and (3) it affects the acetylation of a large number of metabolic proteins. Studies have demonstrated that practically all enzymes are involved in glycolysis, gluconeogenesis, TCA cycle, fatty acid metabolism and glycogen metabolism and they are acetylated, demonstrating the latter effect of butyrate and other HDAC inhibitors on metabolism [138,139].

Butyrate is essential to maintaining the stability of the intestinal barrier because it acts directly on the epithelial cell’s transcriptional regulation. Unfortunately, butyrate intake is decreasing as the quantity of fiber in the human diet lowers [140]. It is well known that the development and it is well known that the integrity of the blood-brain barrier is mandatory for the healthy development and proper functioning of the brain. Thus, the influence of dietary supplementation with FOS in db/db mice (mice phenotypes that are differentiated by the spontaneous mutations in different sites of leptin receptors) on BBB integrity showed an improvement in the hypothalamus [141].

Both epidemiological and observational studies have looked at the effects of a high-fiber diet on memory and cognitive function. Diet and/or microbiota are altered in this research to enhance brain function. Children who eat a high-fiber diet, for example, have superior attentional performance (e.g., working memory, multitasking and staying focused) compared with those who eat a low-fiber diet [115,138,142].

Recently, Shi et al. discovered cognitive deficits (memory of object location, temporal order, etc.) based on a mouse model of long-term dietary fiber deficiency, similar to a low-fiber diet in humans. In addition, intestinal dysbiosis decreased the production of SCFAs and some neuro-inflammatory processes have been highlighted [143]. In another study that enrolled 47 volunteers, the consumption of oligofructose-enriched inulin induced an increasing episodic memory [144].

Studies published in recent years have shown that neurodegenerative diseases can be linked to specific changes in the gut microbiota and the presence of fiber in the diet does not seem to be negligible. Thus, Subash et. al. [145] proved in a transgenic mouse model of Alzheimer’s disease that a diet plenty of fiber, phenols and antioxidants from palm fruits decreased beta-amyloid and improved learning and memory.

Although until recently, dietary fiber intake did not appear to correlate with symptoms of GI or treatment for Parkinson’s [146], recent studies suggest the opposite. The arguments about the beneficial uses of *per os* sodium butyrate in animal models for Parkinson’s disease were already published. Moreover, it is thought that a microbiota that would generate butyrate locally could be much more efficient [147] and a careful selection of prebutyrogenic fiber (or new strategies of gut-microbiota-based) appears that it is already the subject of recent studies [148,149].

Activation of NGI by FOS supplementation could reverse the development of HFHS-induced anxiety-depressive phenotypes. The HFHS diet is known to induce high corticosterone secretion in mice [150]. FOS supplementation corrects this hypersecretion (causing a 36% decrease) and improves HFHS-induced anxious-depressive phenotypes. Therefore, NGI induction could be a common regulator of metabolic and emotional functions of the hypothalamus [127,151,152].

Moreover, Bimuno^®^-galacto-oligosaccharides (B-GOS) supplemented diet (daily for three weeks) reduced the waking cortisol response and altered emotional bias in healthy volunteers compared to a placebo (maltodextrin), while no effects seemed to be induced by FOS [153]. The non-digestible GOS administration for three weeks in mice that previously received a unique lipopolysaccharide dose reduced anxiety, probably by diminished interleukin-1β. The chronically stressed mice also experimented with anxiolytic and antidepressant effects due to GOS and FOS administration [141].

In the battle against mood disorders, a dietary and micronutrient strategy appears useful. Neuro-nutrition is a medical specialty that focuses on improving the nervous system’s and psyche’s performance. This makes use of macro and micronutrients, as well as complementary neuroscience methods. Prebiotic fiber, such as FOS or GOS, are the most often utilized psychobiotics currently, as they help the microbiota function properly on a daily and regular basis. They could offer lower anxiety responses and increase stress adaptability [140].

## 7. Dietary Fiber in the Diet and the Risk of Cardiovascular Disease

Soluble dietary fiber increases the viscosity of the intestinal lumen and slows the absorption of fat and cholesterol, as well as increasing the excretion of bile acids and SCFAs actually results from the fermentation of soluble dietary fiber in the large intestine, and it help prevent coronary heart diseases [154,155].

Fiber decreases LDL-cholesterol, reduces the likelihood of stroke and type 2 diabetes and prevents and cures obesity. Fiber dilutes calories in a meal, delivering long-lasting satiety. One of the risk factors for cardiovascular disease is obesity. To prevent cardiovascular diseases, American Heart Association and other similar groups throughout the globe advocate for a diversified diet that emphasizes plant-based foods, such as fruits, whole grains, oilseeds, vegetables and legumes. Clinical studies that indicate a significant reduction in cardiovascular risk in people who consume a lot of fiber and whole grains than those with a low fiber diet [156,157].

Natural dietary fiber contribute to cardiovascular protection and other physiological effects, such as lowering blood pressure and reducing inflammatory markers. On the other side, no association could not be established between the risk of cardiovascular disease and supplementation with zinc from NDF, even if this ion appears to reduce cardiovascular risk and mortality from coronary heart disease, notably in Mediterranean populations [158,159]. In fact, it has been reported that approximately 40% of patients present natural causes in the development of cardiovascular disease [160]. 

A dose-response meta-analysis examined relationships between total fiber consumption, found in whole grains, fruits and vegetables and heart failure (the latter quantifying both stroke and coronary stroke). According to the findings, increasing total fiber intake by 7 g per day reduces cardiovascular and coronary risk by 9%. Increased insoluble fiber intake in cereals, fruits and vegetables was also linked to a decreased cardiovascular risk. The insoluble fiber in grains and vegetables has also been demonstrated to lower the risk of coronary artery disease. 

The study’s findings apply exclusively to dietary fiber consumption, not dietary supplements [156]. Other chemicals found in cereals and plant products have a cardioprotective impact (lignans, antioxidants, phytosterols, etc.). Other NDF effects are thought to contribute to cardiovascular protection, such as lowering blood pressure, especially in the elderly and reducing inflammatory markers (C-reactive protein) and it confirms the relationship between dietary fiber and coronary heart diseases. 

For example, prospective studies in the US and Europe have shown that a 10 g/day increase in NDF reduces the risk (by 14%) and death (by 27%) of coronary heart disease [161]. Hypercholesterolemia (easy to measure in plasma) is a key risk factor for the incidence of cardiovascular disease. Processed fiber has been the subject of several studies on reducing total cholesterol and LDL-cholesterol, and it has been found that some fiber may have a lowering effect on lipidemia mainly by limiting intestinal absorption of dietary lipids. 

Oat bran, fruits and vegetables are good sources of NDF. According to a meta-analysis of prospective studies, high NDF consumption lowers the risk for heart disease [162]. Several investigations have confirmed the inverse association between fiber and coronary heart disease, corroborating these assumptions. Prospective studies in the United States and Europe have found that increasing NDF by 10 g per day reduced the incidence of coronary heart disease by 14% and mortality from coronary heart disease by 27% [161].

Hypercholesterolemia (abnormally high levels of cholesterol in the blood) is a major risk factor for cardiovascular disease. Several investigations on dietary fiber and the decrease of total cholesterol and LDL-cholesterol levels have revealed that certain fibers may have a reducing effect on lipidemia, mostly via decreasing natural lipids absorption in the intestine. NDF may be found in oat bran fruits and vegetables [163,164].

A high-fiber diet reduces cardiovascular risk factors, decreases LDL cholesterol and raises HDL blood cholesterol levels [165]. Diastolic blood pressure was also reduced; however, there was no significant impact on systolic blood pressure, but there was a tendency to decrease. However, there are insufficient data to distinguish between the effects of the two dietary fiber categories (soluble and insoluble) [166]. The most studied soluble dietary fiber also seem to be the most effective [167].

## 8. Autoimmune Diseases

Autoimmune diseases are chronic conditions with an incidence of 5–10% worldwide, affecting the quality of life of the patients concerned, which can lead to disabilities. More than 80 different autoimmune diseases can affect all body systems. Still, chronic inflammation remains a common feature of autoimmune diseases despite their heterogeneity and generates significant oxidative stress, which results in tissue damage. Autoimmune diseases are characterized by losing the body’s immune tolerance to its constituents and may be classified into organ-specific, MS (multiple sclerosis), T1D (type 1 diabetes) and systemic, PR (rheumatoid arthritis) and SLE (systemic lupus erythematosus) [168].

The content of the food has a vital role in improving people’s lives with autoimmune illnesses and the nutritional condition is essential in the regulation of the immune system. The association between the gut microbiota and systemic immunological responses, especially responses to autoimmune disorders, has gotten a lot of interest in the field of immune-mediated diseases pathogenesis. The intestinal microbiota regulates the humoral and cellular components of the intestinal immune response and reduces chronic inflammation [169].

A daily intake of dietary fiber helps to reduce the absorption of carbohydrates and lipids, reducing the risk of hyperlipidemia. In addition, dietary fiber improves intestinal transit and increases the feeling of fullness. NDF intake is strongly recommended in patients with lupus to limit the onset of metabolic disorders, such as dyslipidemia, which is very common during the disease. It is suggested to take 14 g of fiber per 1000 kcal. 

Numerous studies have shown that there is even an inverse relationship between fiber absorption and the severity of disseminated lupus erythematosus. However, consuming too much fiber can decrease the absorption of certain minerals, proteins and vitamins essential in the management of this disease [170]. Type 1 diabetes (T1D) is an autoimmune disease in which the pancreas’ insulin-producing cells are systematically destroyed. Although type 1 diabetes has a substantial hereditary component, the prevalence of type 1 diabetes has been steadily increasing in recent decades, suggesting that environmental variables (hygiene, antibiotic usage and food) have a significant role.

SCFAs, which are generated in considerable amounts in the colon by digesting NDF, are the primary metabolites of the intestinal microbiota. These compounds increase the number and function of induced regulatory T cells in the colon. In addition, acetate promotes intestinal barrier function, while butyrate is well known for its anti-inflammatory effects. Acetate and butyrate-producing diets have improved intestinal integrity and decreased serum diabetic cytokine levels, such as IL-2.1 [168]. In type 1 diabetes, intestinal dysbiosis increases intestinal permeability and immune-regulatory changes can trigger the autoimmune response that leads to the destruction of β cells in the pancreatic islets [171].

Dietary therapy can positively influence on autoimmune illnesses, and this is an option to explore when used with traditional treatment to treat autoimmune disease symptoms. Irritable bowel syndrome (IBS) is a disorder that is becoming increasingly widespread among people. The pathophysiology is multifactorial, with peripheral (digestive disorders, intestinal micro-inflammation, intestinal permeability disorders and the role of the microbiota) and central factors (visceral hypersensitivity, bone marrow control abnormalities and psychological factors) of the diet also having significant influence. 

For the diagnosis of irritable bowel syndrome, the associated symptoms must have been present for at least six months. In addition, there must be a criterion for the frequency of abdominal pain, at least one day a week for the last three months. There are different subtypes of IBS depending on the dominant type of transit, and thus we can talk about irritable bowel syndrome with predominant diarrhea (IBS-D), with predominant constipation (IBS-C) or with alternative episodes of diarrhea—constipation (mixed forms, IBS-M) can also be unclassified forms [171].

One of the keys recognized hygienic and nutritional suggestions in managing chronic constipation is to increase NDF intake. Soluble and insoluble fiber are two types of NDF. The model of soluble fiber is psyllium, whereas the paradigm of insoluble fiber is wheat. Adequate fiber intake (above 25 g/day) has been proven to reduce the risk of chronic constipation in women and the elderly [172]. Irritable bowel syndrome patients have always been advised to have a high-fiber diet. Dietary fiber can help with constipation in a variety of ways. Increasing NDF consumption (to 20–40 g/day) must be done gradually, in ten days [173].

In a 2021 clinical trial involving 40 patients (15 women and 25 men) for a consumption of 30 g/day of high-fiber wheat bran, a significant change in hard stool consistency was observed constipated subjects, as well as relief of symptoms associated with transit dysfunction of the digestive system [174]. Insoluble NDF promote bowel movement and transit by mechanically stimulating and irritating the colonic mucosa, resulting in increased secretion and colonic peristalsis. In addition, NDF that form high-viscosity colloidal dispersions (e.g., psyllium) are partially fermented and form a gel that normalizes stool formation. 

In contrast, soluble NDF are fermented by bacteria in the large intestine, resulting in fermentation by-products, such as gases (carbon dioxide and methane) and SCFAs [175,176,177]. According to an increasing body of research, NDF appears to affect the makeup of the gut microbiota. SCFAs are fermentation-derived compounds that lower luminal colonic pH and encourage the development of beneficial bacteria, such as *Lactobacillus* sp. and *Bifidobacteria* sp. In addition, butyrate, formed when dietary fiber is fermented, reduces colon inflammation by inducing T cell apoptosis and suppressing interferon-mediated inflammation-γ (IFN-γ) [177].

Natural dietary fiber’s connection with the neuroendocrine system of the intestinal system is increasingly being studied. The enteric nervous system and the gastrointestinal endocrine cells compensate the neuroendocrine system of the gastrointestinal tract. At the epithelium level, many kinds of endocrine cells can be present. Endocrine cells account for around 1% of all epithelial cells in the gastrointestinal system and contain specific sensors that release hormones in response to stimuli [178]. Each cell type secretes one or many messenger particles. 

**Figure 5:**
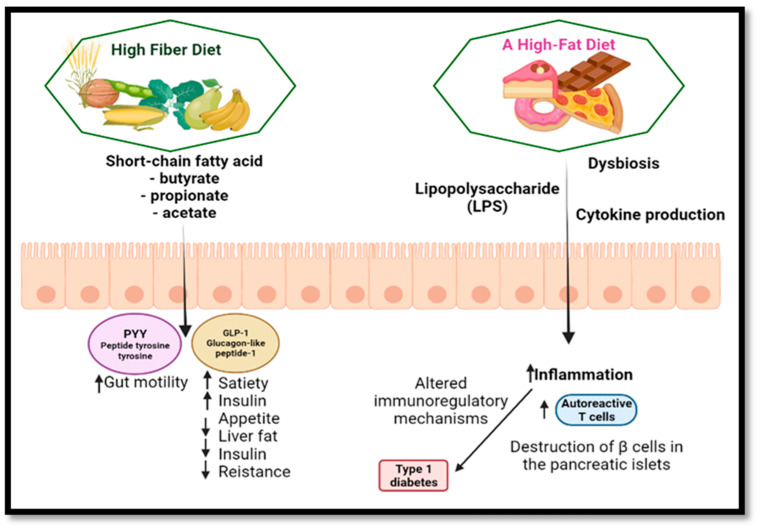
The consequences of a high-fiber diet vs. a high-fat diet. Created with BioRender.com.

The gastrointestinal tract’s neuroendocrine system controls various processes, including local immune defense, motility, absorption, secretion and food consumption [179]. The hormone serotonin, which is known to play a critical role in visceral sensitivity, maybe stimulated by changes in luminal colon pH. Several intestinal hormones, including YY (PYY) peptide and glucagon-1-like peptide, which enhance insulin release, promote satiety and decrease hunger, appear to be affected by SCFAs generated by processed fiber fermentation (Figure 5) [180,181]. PYY is known to increase water and electrolyte absorption. PYY also suppresses prostaglandin E_2_ production. This explains why processed fiber has an impacts on gastrointestinal transit and secretion [177].

Recent research comparing NDF administration to placebo indicated that fiber supplementation (particularly with psyllium) was beneficial in alleviating overall IBS symptoms in 14 randomized clinical trials comprising 906 people with IBS [181]. The fiber supplementation appears to be safe, albeit bloating and temporary stomach dilatation may occur if it is introduced too quickly into the diet [177].

## 9. The Role of High-Fiber Diets in Cancer Prevention

Dietary variables have a part in cancer prevention as well. Obesity, sedentary behavior, poor nutrition, smoking and excessive alcohol intake are linked to cancer. Adopting a balanced diet rich in fruits and vegetables is best to avoid cancer. This diet has been demonstrated to have a preventive impact against cancer, particularly colon and breast cancer. Increased NDF consumption has been linked to a substantially reduced risk of colorectal cancer in clinical investigations [182]. In the case of people with low fiber intake, it is estimated that doubling the fiber intake can reduce the incidence of colorectal cancer by more than 35% [183]. The protective effect of NDF against colorectal cancer is manifested by several mechanisms [184,185]:

- insoluble fiber increases the mass of the fecal bowl, increase intestinal peristalsis and reduce the contact time of toxic compounds with the intestinal mucosa;
- soluble fiber in particular, but also resistant starch, modify the fecal microorganisms and increase the number of saprotrophic bacteria, which exert a beneficial effect on the intestinal microbiome by SCFAs generated by the fermentation process while causing a decrease in pH, thus inhibiting pH-sensitive, potentially pathogenic bacterial species that could lead to potentially carcinogenic compounds;
- production of butyrate by fermentation of soluble fiber as well as resistant starch delays the proliferation of malignant cells, reduces inflammatory processes and promotes DNA regeneration;
- reducing the percentage of body fat, contributes to the decrease of estrogen secretion due to decreasing in the number of secretory adipocytes. The consumption of NDF protects against breast and endometrial cancer by binding estrogen to the colon and increasing its fecal elimination. Along with fiber and other constituents of vegetables, fruits and whole grains protect against cancer, especially compounds with an antioxidant action (flavones, polyphenolic compounds, anthocyanins, carotenoid pigments, etc.) [182].

The cell biology of cancer is highly complex, and the appearance of the disease involves a long chain of changes that occur in various cellular processes. The mutations that occur accumulate over time, it is not enough just a mutation for malignant cells, the process is highly complex and takes place over time. Initially, there is an overexpression of oncogenes and inhibition of tumor suppressor genes [186].

Nearly 40% of cancers can be attributed to behavior that requires avoiding major risk factors, such as smoking cessation, reduced alcohol consumption, physical activity, reduced inactive lifestyle, reduced body weight and a varied and balanced diet. Therefore, prevention is an essential means in the fight against cancer and involves avoiding favorable factors: ultraviolet radiation, biological risks, viruses or bacteria, exposure to carcinogens contained in smoke, tobacco, alcohol, arsenic, benzene, asbestos [138,187].

Butyrate, the salt of SCFA resulting from the fermentation of fiber by anaerobic bacteria, is being studied in the colon cancer pathology because it could be used as a chemo-preventive agent for colon cancer. It is the primary fuel source for colonocytes and is used preferentially over glucose or other SCFAs. On the other hand, the butyrate also induces cell apoptosis [138], while regular fiber consumption significantly reduces the risk of colorectal cancer [115].

**Figure 6:**
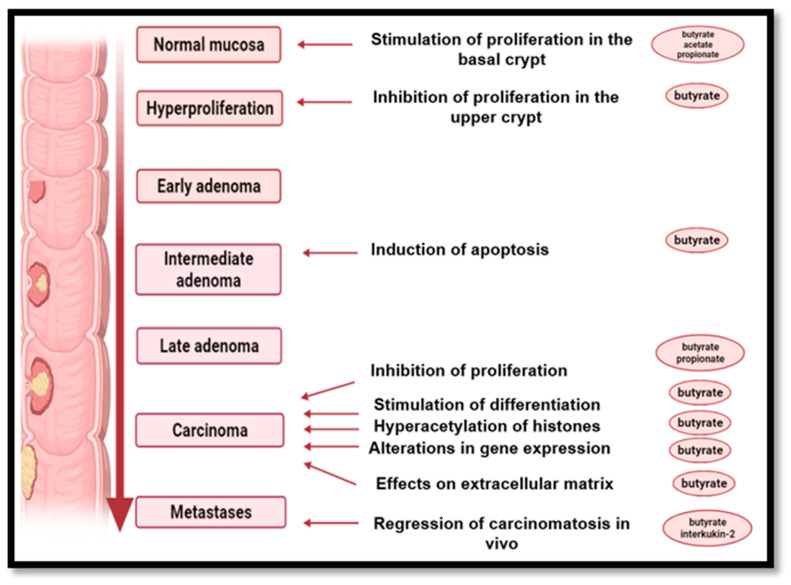
The impact of short-chain fatty acids on the mucosa of the colon. Created with BioRender.com.

In an in vitro study in which a sample of colonic tissue was incubated with deoxycholic acid, it was observed that immune-histochemical measurement with bromodeoxyuridine resulted in cellular hyper-proliferation. At the same time, the butyrate antagonized deoxycholic acid-induced hyper-proliferation in colonic tissue in the same experimental conditions. In addition, butyrate-induced cell growth limitation has been observed in several cell cultures from human carcinomas LIM-1215, SK-CO-1, HT-29, HRT-18 and SK-CO-1 (Figure 6) [115,188,189].

Oral, pancreatic and colon cancer have been linked to *Fusobacterium nucleatum*, one anaerobic Gram-negative bacteria. This is one of the most prevalent bacteria in the mouth, and some research suggests that this Gram-negative bacterium is linked to oral inflammatory illnesses, including periodontitis and gingivitis. The results show that *Fusobacterium nucleatum* levels are significantly higher in tumor tissues and stool samples of colorectal cancer patients than in healthy patients. *Fusobacterium nucleatum* may contribute to the development of tumor processes and is considered to be a potential risk factor for tumor progression [190,191].

The presence of this bacterium was associated with the Western diet rich in red meat, semi-prepared foods and desserts, while its absence was correlated with a diet rich in fiber and whole grains. Thus, diets high in fat and low in fiber increase the chances of developing colorectal cancer and also the proliferation of *Fusobacterium nucleatum* [192,193].

Alcohol appears to be a warning cancer risk factor. High levels of circulating androgens are associated with a higher risk of hormone-dependent cancer linked to alcohol usage. This association could be modulated by NDF intake because experimental studies have shown that fiber has increased levels of sex hormone-binding globulin (SHBG). This process causes a decrease in serum steroid hormones. Fiber intake could modulate the association between alcohol consumption and the risk of hormone-dependent cancers [194].

The risk factors that play a key role in developing or preventing cancer are diet, alcohol consumption, smoking and reduced physical activity [195,196]. There is a strong association between an obese diet and cancer risk [197]. On the other hand, multiple meta-analyses have found that eating fiber or high-fiber diets (fruits, vegetables and whole grains) lowers cancer risk [198,199,200]. As a result, several studies have found that eating a high-fiber diet reduces cancer risk (gastric, pancreatic, colorectal, prostate). As cancer cell biology is exceedingly complicated, drawing firm conclusions is quite difficult [201,202,203,204].

## 10. Dietary Fiber Consumption Restrictions

Excessive NDF intake can lead to bloating (flatulence), diarrhea or intestinal obstruction in people who do not consume sufficient fluids. In some digestive system diseases, such as Crohn’s disease or intestinal obstruction, fiber intake must be limited and under medical supervision [205]. Patients suffering from malabsorption should be advised to eat NDF with caution. Careful fiber consumption is also recommended for patients who have undergone bowel surgery. The higher the fiber intake, the higher the water intake should be because there is a risk of dehydration. Health professionals recommend a diet rich in high-fiber products. 

Although this recommendation is suitable for most patients, some people should not overdo such a diet. The Phytobezoar is a dense mass of NDF, seeds, leaves or other pieces that collects in the stomach or small intestine, obstructing the region. Some people have a predisposition to Phytobezoar. For example, excessive consumption of foods high in fiber combined with improper chewing of food enhances Phytobezoar formation that can be amplified by the deposition of fats, salt residues and fiber. In addition, aging causes a decrease or even loss of intestinal elasticity, the resulting constipation can be considered to be a favorable factor in the formation of intestinal Phytobezoars. 

Finally, the small intestine obstruction resulting from tumors and inflammation is also considered in terms of intestinal Phytube appearance. Thus, patients with intestinal obstruction should consume a balanced intake of fiber without excessive consumption [206]. The incidence of intestinal diseases has increased in recent years. Young people are increasingly affected, and it is likely that intestinal diseases will become a major health problem shortly, even in developing countries. However, the cause for their appearance is not entirely clear. 

For example, in Crohn’s disease and ulcerative colitis, both chronic inflammatory bowel diseases (IBD), no clear explanation has been found for their occurrence. The genetic predisposition or even the geographical area may be attributed to the prevalence of these diseases. A high-fiber diet (and fat-poor) versus a modified-standard American diet (which included higher quantities of fruits, vegetables and fiber) was studied in patients with ulcerative colitis. 

The first diet increased the relative abundance of *Bacteroidetes* and *Faecalibacterium prausnitzii* in fecal samples, while the *Actinobacteria* relative abundance decreased. At the same time, the inflammation markers (for example, the acetate level) increased in both diets compared with control. As a result, dietary fiber diets could be beneficial in patients with ulcerative colitis in remission, especially since all enrolled patients remained in remission throughout the study [207].

However, the administration of dietary fiber in food patients with inflammatory bowel disease needs more randomized controlled trials on adult populations and even more in pediatric ones before being widely recommended [208].

As the association between fiber intake and IBD development has not been clearly beneficial or not, fiber consumption remains controversial, although many articles show a positive effect on NDF intake [194,209]. In addition, there is no such thing as a perfect diet, many foods, even those considered healthy, such as fresh vegetables and fruits, can trigger a Crohn’s attack or aggravate symptoms and a long-term excess of NDF can disrupt the absorption of macro and micronutrients [210].

## 11. Conclusions

Dietary fibers are important nutrients that provides many benefits to the body, no energy value and are necessary for the normal functioning of the digestive tract and the whole human body. They are not digested or absorbed but instead help the intestinal tract function optimally and contributes to the body’s detoxification. The main role of dietary fiber is to prevent constipation and promote intestinal motility regulation. Fiber is also crucial in diets to lose weight, as it gives a feeling of long-term satiety, and at the same time, plays an important role in preventing metabolic diseases. 

Furthermore, clinical data indicate a beneficial effect of a fiber-enriched diet on neurodegenerative diseases. In this direction, we need more clinical data and evaluations to confirm and highlight the mechanism of action of fiber on the central nervous system. Some reports indicated a positive effect of fiber on the behavior of children with autism, for example. Clinical trials in this direction are still insufficient to have a complete and definite picture of the mode of action of dietary fiber. However, when the fiber level increases, the fluid level must also be increased, as the fiber absorbs water from the intestine. Therefore, consuming various fruits, vegetables and grains covers the daily amount of fiber needed.

It should be noted, however, that excessive fiber consumption can cause digestive symptoms, such as flatulence and abdominal cramps or may affect the absorption of essential minerals from foods, such as iron, zinc or calcium.

## Figures and Tables

**Table 1:** The classification of dietary fibers according to the composition, properties and main sources [13,14,15,16].

Poly-/Oligo-Saccharides Class	Sources	Main Units	References
Non-Starch Polysaccharides, MU ≥ 10			
Cellulose *	Cereals, pulses—outer layers, root and leafy vegetables, legumes, pears and apples	Glucose monomers	[17]
Hemicellulose **	Cereal bran and whole grains (starchy endosperm and aleurone layer), vegetable and fruit cell walls	D-xylose, D-mannose, D-galactose and L-arabinose	[14]
Mannans and heteromannans	Date, green coffee bean seeds and aloe vera Grain legumes (endosperm) Iris seeds and lily bulbs Norway spruce wood pulp	Mannans *, galactomannans ^#^, glucomannans ^#^ and galactoglucomannans	[16]
Pectins ^#^	Apple and citrus peel (and other fruits), cabbage, whole grains, beetroot and grain legumes	Arabinose, rhamnose, galactose sugars and galacturonic acids	[14]
Gums	Xanthan gum * Alginates ** Agar-agar ** Carrageenan **	Pentose and hexoze monomers	[18]
Mucilages ^#^	Aloe vera, Cactus, Okra, Hibiscus	Main glycoproteins	[16]
Inulin ^#^ and fructans	Jerusalem artichoke, Chicory root, onion and cereal grains	fructofuranosyl residues	[15]
Non-Digestible (resistant) oligosaccharides, MU = 3–9			
α-galactosides **	Chickpea, bean, lentil, etc.	Raffinose, stachyose, verbascose	[15]
β-fructo-oligosaccharides **	Polymers resulted from polysaccharides hydrolysis (inulin and lactose hydrolysis produce FOS and, respectively, GOS).	β-Fructo- (FOS), α-galacto- (GOS), β-galacto- (TOS), xylo- (XOS), arabino-xylo- (AXOS) oligosaccharides	[15]
Resistant dextrins **	cereal-based vegetable milk, baked goods, dairy products and granola bars	Poly-D-glucose	[15]
Polydextrose **	Cakes, candies, mixes and frozen desserts and beverages	Poly-D-glucose	[15]
Resistant Starches * (RS), MU ≥ 10			
RS type 1	Grains and legumes (whole or partially milled)	physically inaccessible starch	[15]
RS type 2	High-amylose starches, green bananas	granular starches	[15]
RS type 3	Cooled starches in cooked starchy foods and enzyme-debranched starches	gelatinized and retrograded starches	[19]
RS type 4		chemically modified (mainly cross-linked starches)	[15]
Associated Substances Non-carbohydrates			
Lignin *	Fruits, particularly strawberries and peaches	Coumaryl, coniferyl and sinapyl alcohols (aromatic alcohols)	[17]
Waxes *	Wax is present in rice bran, seed and seed hulls of sunflower	Long alkyl chains	[20]
Chitins *	Fungus’ cell walls, lobster, crab and shrimp exoskeletons and insects	N-acetylglucosamine	[15]
Phytates/Phytic acid ^#^	Plant seeds, mainly in legumes, peanuts, cereals and oilseeds and generally found in almost all plant-based foods	-	[21]

* Insoluble, ** Slightly soluble, ^#^ Soluble; and MU = monomeric units.

**Table 2:** Studies reporting fiber administration in humans.

Study Type	Nature of Participants	Duration Administered	Type and Dose of Fiber Administered	Main Findings	References
Randomized, double-blind parallel-group design controlled trial	Hypercholesterolemic adults	6 weeks	6 g concentrated β-glucan/day	Reduced total and LDL cholesterol	[93]
Randomized, single-blind, controlled, crossover intervention trial	Impaired glucose tolerance participants	18 weeks	15 g arabinoxylan/day	Improved fasting serum glucose	[94]
Randomized, single-blind, controlled, crossover intervention trial	Healthy and glucose intolerant subjects	one year	6 g of fiber partially hydrolyzed guar gum with each meal	Reduced postprandial plasma glucose, postprandial insulin, triacylglycerol levels	[95]
Randomized, single-blind, controlled, crossover intervention trial	Pre and post-menopausal, hypercholesterolemic women	6 weeks	5 g psyllium/day	Reduced total cholesterol for post-menopausal women but not in pre-menopausal women	[96]
Randomized, crossover, a single-blind, dietary intervention	Free-living subjects	28 days	Control diet: 25 g dietary fiber/day; LKFibre * diet: 55 g dietary fiber/day	Reduced total and LDL cholesterol	[97]
Clinical trial study	Healthy subjects; type-2-diabetes and pre-diabetics subjects.	16 weeks	10 g/day gum Arabic	Decrease in glycosylated hemoglobin (HbAc1), decrease fasting blood glucose, health improvement	[98]

* LKF—lupin kernel fibers.

## Data Availability

Not applicable.
